# Anomalous excess noise behavior in thick Al_0.85_Ga_0.15_As_0.56_Sb_0.44_ avalanche photodiodes

**DOI:** 10.1038/s41598-023-36744-7

**Published:** 2023-06-19

**Authors:** Harry I. J. Lewis, Xiao Jin, Bingtian Guo, Seunghyun Lee, Hyemin Jung, Sri Harsha Kodati, Baolai Liang, Sanjay Krishna, Duu Sheng Ong, Joe C. Campbell, John P. R. David

**Affiliations:** 1 Department of Electronic and Electrical Engineering, University of Sheffield, Sheffield, S1 3JD UK; 2 Department of Electrical and Computer Engineering, University of Virginia, Charlottesville, VA 22904 USA; 3 Department of Electrical and Computer Engineering, The Ohio State University, Columbus, OH 43210 USA; 4 California NanoSystems Institute, University of California, Los Angeles, CA 90095 USA; 5 Faculty of Engineering, Multimedia University, 63100 Cyberjaya, Malaysia

**Keywords:** Optical sensors, Optoelectronic devices and components

## Abstract

Al_0.85_Ga_0.15_As_0.56_Sb_0.44_ has recently attracted significant research interest as a material for 1550 nm low-noise short-wave infrared (SWIR) avalanche photodiodes (APDs) due to the very wide ratio between its electron and hole ionization coefficients. This work reports new experimental excess noise data for thick Al_0.85_Ga_0.15_As_0.56_Sb_0.44_ PIN and NIP structures, measuring low noise at significantly higher multiplication values than previously reported (*F* = 2.2 at *M* = 38). These results disagree with the classical McIntyre excess noise theory, which overestimates the expected noise based on the ionization coefficients reported for this alloy. Even the addition of ‘dead space’ effects cannot account for these discrepancies. The only way to explain the low excess noise observed is to conclude that the spatial probability distributions for impact ionization of electrons and holes in this material follows a Weibull–Fréchet distribution function even at relatively low electric-fields. Knowledge of the ionization coefficients alone is no longer sufficient to predict the excess noise properties of this material system and consequently the electric-field dependent electron and hole ionization probability distributions are extracted for this alloy.

## Introduction

Avalanche photodiodes (APDs) are widely used in some photon starved applications such as LiDAR, gas sensing and 3D mapping. This is due to their internal gain, which can improve the signal-to-noise ratio of a detector system by reducing the impact of the readout electronic noise. APD gain results from the process of impact ionization, which is stochastic and therefore generates its own noise, referred to as ‘excess noise’. The excess noise factor of an APD, *F*, usually increases with gain and is the limiting factor in the useful performance of APDs. According to the simplified local model of avalanche multiplication, *F* is related to the ratio between the hole and electron ionization coefficients (*β* and *α*, respectively). A small ratio results in reduced excess noise when electrons initiate the ionization process^1^. This ratio of *β*/*α* is referred to as *k* and this is a fundamental property of the avalanching material. A smaller *k* value also results in APDs with higher gain-bandwidth product, because the effect of carrier feedback is minimized^2^. *F* is approximately related to multiplication factor and *k* by Eq. (1)^1^:

1 $$F = kM + \left( {2 - {\raise0.7ex\hbox{$1$} \!\mathord{\left/ {\vphantom {1 M}}\right.\kern-0pt} \!\lower0.7ex\hbox{$M$}}} \right)\left( {1 - k} \right)$$

where *M* is the mean avalanche multiplication factor. In order to minimize *F* when electrons initiate the ionization process there has been considerable effort to find materials where *k* is very small. For wavelengths below 1000 nm, silicon is an excellent material for APDs, capable of providing very low *F* and high gain values. For wavelengths beyond 1000 nm in the short-wavelength infrared (SWIR) region, the current commercial generation of room temperature APD detectors use almost exclusively an In_0.53_Ga_0.47_As (InGaAs) absorption region with an InP or InAlAs multiplication region on a InP substrate. As these multiplication materials have values of *β* and *α* that are not very disparate, the *F* increases rapidly as *M* increases, limiting the maximum sensitivity of these InGaAs APDs. Recently there has been considerable interest in AlGaAsSb alloys capable of lattice matching on InP substrates^3–8^ for use as the multiplication region of an APD. While AlAs_0.56_Sb_0.44_ (AlAsSb hereafter) exhibits an extremely low *k* and very low excess noise^3,4^, its high aluminum content results in high surface dark currents unless passivated. The addition of small amounts of gallium to AlAsSb forming Al_0.85_Ga_0.15_As_0.56_Sb_0.44_ (Al_0.85_Ga_0.15_AsSb hereafter) improves the surface stability and decreases the temperature sensitivity of avalanche multiplication^9,10^ of the material, while still maintaining lattice match to InP.

Initial AlGaAsSb studies investigated thin avalanching structures^8,11^ where the reduced excess noise was largely attributed to the effects of the carrier ‘dead space’^12,13^—the minimum distance that a charge carrier must travel before it acquires sufficient energy to impact ionize. More recently, thicker structures have been investigated following studies showing that the *k* of AlAsSb is significantly smaller at lower electric fields^3^. These have included studies of nominally 1-µm PIN structures under pure electron injection conditions grown as a digital alloy (DA)^5^ and a random alloy (RA)^6^. Those measurements were only undertaken up to multiplication values of ~ 16, and the results interpreted according to McIntyre’s Eq. (1). The different background dopings in the structures further complicated the interpretation of their results. Following on from an accurate determination of the ionization coefficients in this material system over a wide electric field range^7^, a comprehensive investigation of the bulk excess noise properties of Al_0.85_Ga_0.15_AsSb PIN and NIP structures with multiplication widths ranging from 390 to 1020-nm has now been completed for the first time. These have been studied using a range of wavelengths to yield pure electron-initiated multiplication, pure hole-initiated multiplication, and variously mixed electron-initiated and hole-initiated multiplication conditions. These measurements show that in thick Al_0.85_Ga_0.15_AsSb avalanching structures, the *F* vs *M* is *not* determined by the ionization coefficient ratio *k* and the conventional McIntyre equation, even with the addition of any carrier dead space^12^. Modelling undertaken here shows that to explain the multiplication and very low *F* seen in this material system, the shape of the ionization probability density function (PDF) has to be significantly different to the simple exponential forms assumed for most other avalanching material systems. The electron and hole PDFs that are capable of fitting the multiplication and excess noise over a wide electric-field range of 400–675 kV/cm in this material are extracted and these can be used for the design of low noise APD structures.

## Wafer and device details

Random alloy (RA) and digital alloy (DA) Al_0.85_Ga_0.15_AsSb PIN structures and a DA NIP structure were grown on semi-insulating InP substrates using molecular beam epitaxy. A DA growth technique using two alternating ternary layers was used to overcome the perceived problem of phase separation in these thick quaternary alloy systems. Details of the structures investigated are shown in Table 1.

**Table 1:** Parameters of Al_0.85_Ga_0.15_As_0.56_Sb_0.44_ wafers used in this study.

Wafer	Growth type	Intrinsic region width, *w* (µm)	*N*_i_ [×10^15^cm^−3^] ± 1 × 10^15^ cm^−3^	*N*_p_ [×10^17^cm^−3^] ± 1 × 10^17^ cm^−3^
PIN1	DA	0.890	15	10
NIP1	DA	0.890	19	10
PIN2	RA	1.020	1.0	20
PIN3	RA	0.590	2.5	20
PIN4	RA	0.390	5.0	20

**Figure 1:**
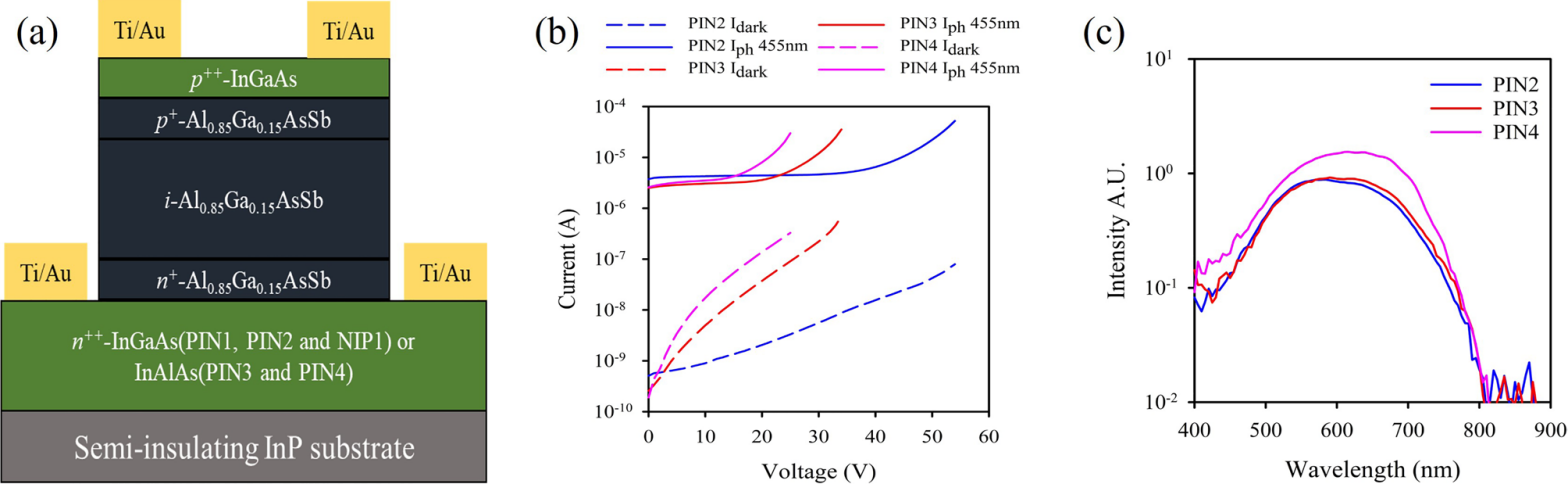
(**a**) A schematic diagram of devices used in this work. (**b**) Photocurrent (455 nm) and dark current on PIN2, PIN3 and PIN4. (**c**) Photoresponse for PIN2, PIN3 and PIN4 at different wavelengths.

PIN1, NIP1 and PIN2 were grown with a highly doped 400–500-nm InGaAs bottom contact layer and a 20-nm InGaAs top contact layer as shown schematically in Fig. 1a. PIN3 and PIN4 were grown with a highly doped 500-nm InAlAs bottom contact layers and 20-nm InGaAs top contact layer. The nominal widths of the Al_0.85_Ga_0.15_AsSb cladding layers were 300-nm for the top cladding and 100-nm for the bottom cladding. The actual intrinsic region widths and dopings were calculated using capacitance–voltage measurements and are detailed in Table 1. Mesa structures with diameters of 420, 220, 120, and 70 µm were fabricated using wet etching in a solution composed of 20 g citric acid:5 ml H_3_PO_4_:5 ml H_2_O_2_:120 ml H_2_O. Ti/Au was used for top and bottom contacts.

## Methodology

Capacitance–voltage measurements were performed at a frequency of 1 MHz using an HP4275A LCR meter. A static dielectric constant of 11.4 was used to determine the depletion width and background doping concentration. Dark current–voltage measurements were performed using an HP4140B picoammeter. Figure 1b shows the reverse dark currents for the three RA grown structures, PIN2, PIN3 and PIN4, together with the bias dependent photocurrent obtained under 455 nm illumination. The wavelength-dependent photocurrent for these structures, measured using a tungsten halogen bulb and a monochromator, (Fig. 1c) shows that the absorption cut-off of these structures is at ~ 800 nm as expected for this composition^14^. 

Excess noise measurements were performed at a centre frequency of 10 MHz using the measurement system described by Lau et al*.*^15^. This system allows excess noise to be measured at high values of multiplication by using a phase sensitive technique to remove any contributions from dark currents. The photocurrent was measured using the transimpedance amplifier of the noise measurement system, and avalanche gain was calculated from this. A baseline correction was used to account for changes in the carrier collection efficiency at electric fields where impact ionization is occurring^16^. Careful calculation of avalanche gain using baseline correction is essential because the accurate calculation of *F* is highly sensitive to small changes in the calculated gain. 

Fibre-coupled LEDs of varying wavelengths were used to illuminate the device to avoid the random intensity noise associated with semiconductor lasers. A wavelength of 455-nm was used for pure carrier injection conditions, where ≥ 98% of photogenerated carriers are generated in the top cladding layer^17^, and 780-nm was used to generate a fully mixed carrier injection profile, where carriers are generated uniformly across the high-field region. 530-nm and 625-nm wavelength LEDs were used to generate intermediately mixed carrier injection profiles.

## Results

**Figure 2:**
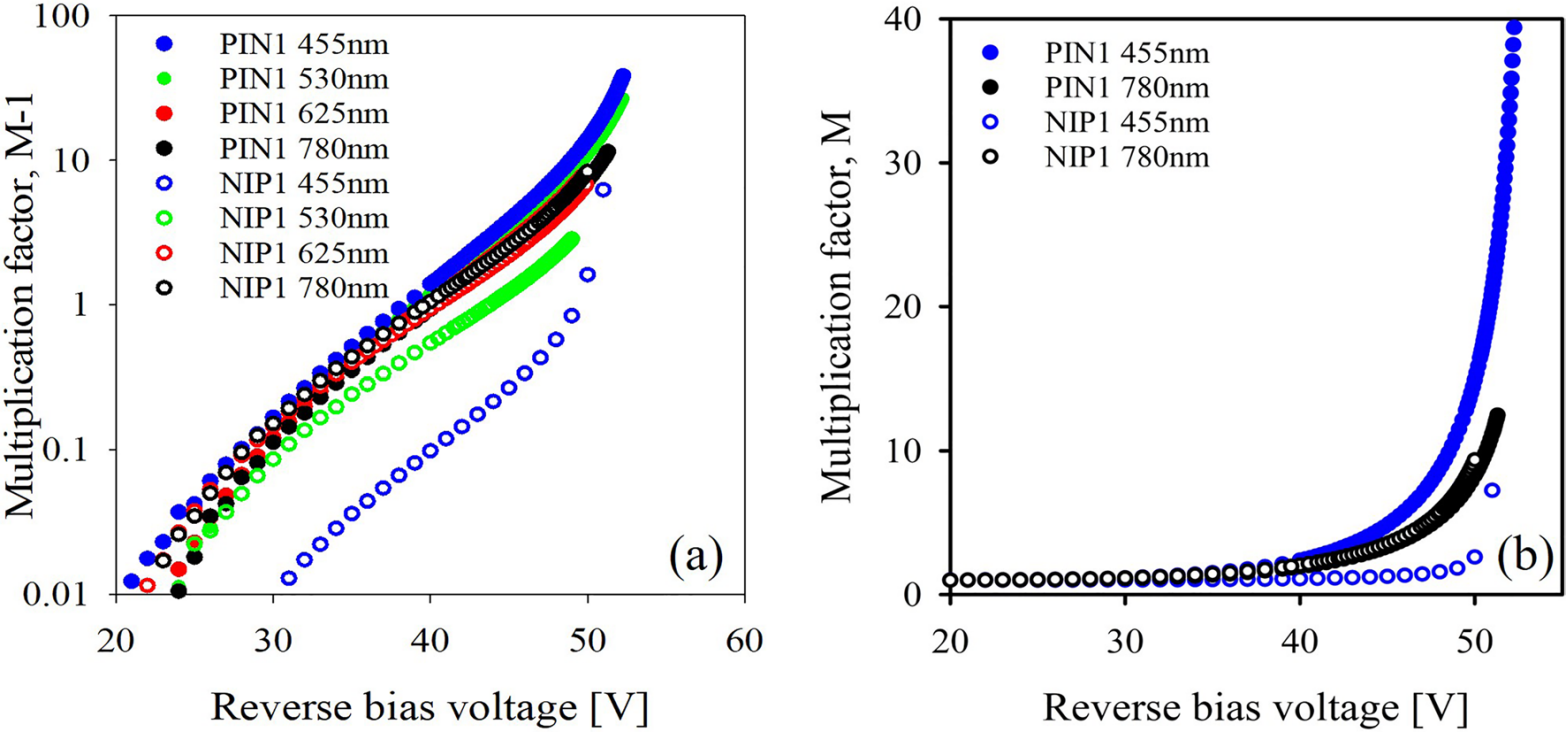
(**a**) Multiplication data for PIN1 and NIP1 under a range of injection conditions, shown in the form *M*-1. Data for NIP1 under 625-nm illumination are omitted for clarity. (**b**) Multiplication for PIN1 and NIP1 under pure and fully mixed injection conditions.

Figure 2a shows multiplication data for PIN1 and NIP1 under varying injection conditions. This data is shown on a logarithmic scale and in the form *M*-1, so that the onset of multiplication can be seen. Multiplication decreases with increasingly mixed injection conditions in the PIN structure and increases with increasingly mixed injection conditions in the NIP structure. The change in multiplication factor under slightly mixed injection conditions (530-nm illumination) is significantly larger in the NIP structure than in the PIN structure. Figure 2b shows multiplication under pure and fully mixed injection conditions for PIN1 and NIP1. The multiplication under 780-nm illumination was almost identical in the PIN and NIP structures and with the slight discrepancy attributed to the small difference in doping between the structures.

**Figure 3:**
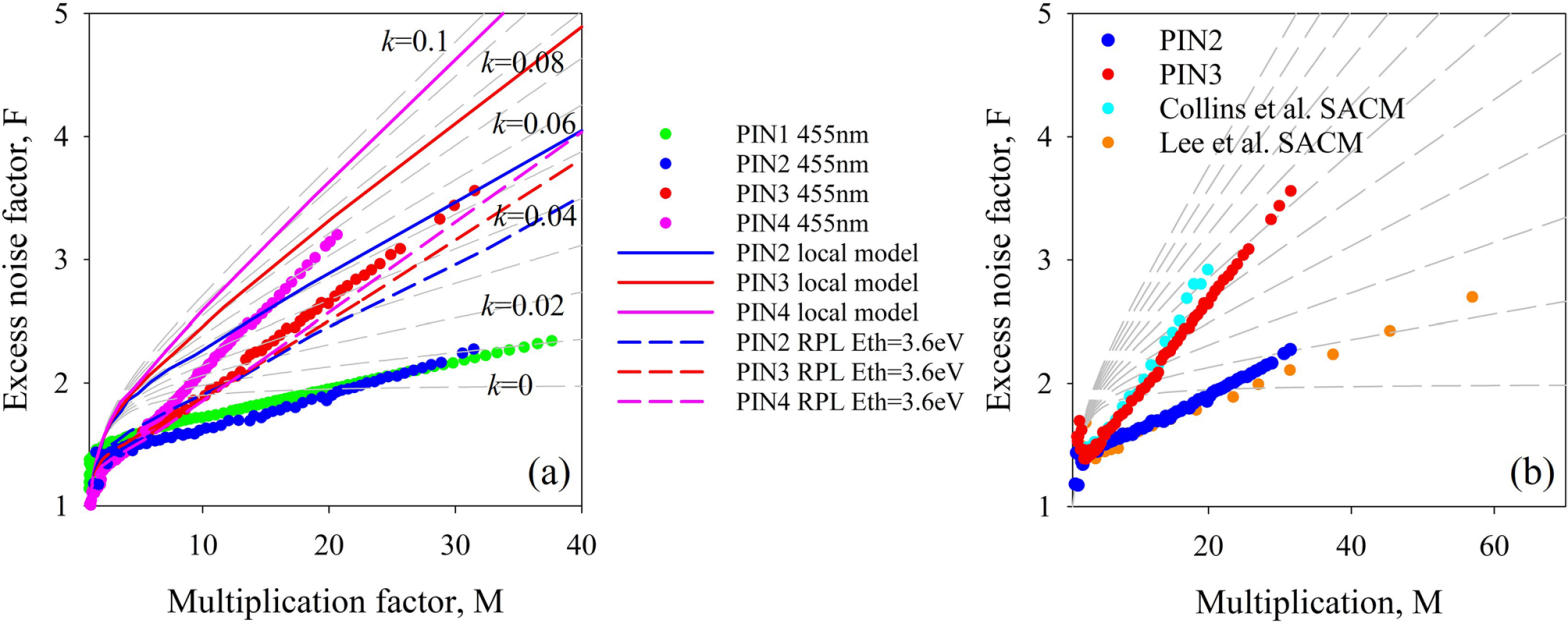
(**a**) Excess noise data for the PIN structures measured in this work. The solid colour lines are the excess noise predicted by the local model and the dashed colour lines are those simulated using an RPL model and ionization threshold energies of 3.6 eV. (**b**) A comparison of excess noise for SACM APDs operated at 1450 nm and 1550 nm^18,19^. Compared to that of PIN2 and PIN3. Dashed lines in both figures indicate the noise predicted by McIntyre’s local model for an effective *k* of 0 to 0.1 in steps of 0.01.

Figure 3 shows pure electron injection excess noise data for each PIN structure. Excess noise increases with decreasing intrinsic region width, reaching an *F* of 2 at multiplication values of 25, 12, and 10 for PIN1 (and PIN2), PIN3 and PIN4 respectively. The excess noise did not vary significantly between the RA and DA structures of similar thicknesses, PIN1 and PIN2, suggesting that despite differences in the growth technique used, the impact ionization characteristics are very similar. The small difference in *F* between PIN1 and PIN2 is probably due to the differences in avalanche widths and background doping between the structures. Although these *F* are obtained using 455 nm light, on structures representing just the multiplication regions, near identical results have been obtained in Al_0.85_Ga_0.15_AsSb using wavelengths of 1450–1550 nm when combined with a low electric field InGaAs^18^ or GaAsSb^19^ absorber as shown in Fig. 3b. The excess noise performance in these APDs is determined by the Al_0.85_Ga_0.15_AsSb high field multiplication regions, estimated to be ~ 600nm^18^ and 1000 nm^19^ and are therefore similar to PIN3 and PIN2. The previously reported results for PIN1 and PIN2 in references^5,6^ were calculated assuming that *F* follows McIntyre’s local model Eq. (1), and significantly overestimate the noise at low values of multiplication. The measurement technique, using a noise figure meter, also limited the maximum multiplication for which excess noise could be reliably measured to ~ 16. In the current measurements an *F* of ~ 2.2 could be obtained at a multiplication of 38 for PIN1, similar to that reported for an AlAsSb structure of similar thickness^4^. Reducing the avalanche region width to 590 nm and 390 nm in PIN3 and PIN4 respectively causes a significant increase in the excess noise measured. The excess noise measured in these PIN structures agrees well with the results reported in full separate absorption and multiplication region avalanche photodiode (SACM-APD) structures with similar multiplication widths^18,19^. A report of lower *F* in a nominally 600 nm PIN^20^ has been attributed to a graded electric field^21^ rather than the constant electric field investigated reported here. The McIntyre equation tends to overestimate the excess noise even in relatively thick structures of many materials such as InP^22^ and InAlAs^23^. However, allowing for a ‘hard’ dead space with a magnitude determined by the carrier threshold energies, followed by an exponential ionization probability, has enabled the measured excess noise to be reasonably replicated using a random path length (RPL) model^24^. Attempts to do something similar with a threshold energy of 3.6 eV for both electrons and holes in Al_0.85_Ga_0.15_AsSb using the ionization coefficients from Guo et al.^7^ manages to reduce the predicted noise from that of the local model, but this still gives a poor fit to the experimental results as shown by the coloured dashed lines in Fig. 3. This suggests that the ionization probability distributions (PDF’s) in Al_0.85_Ga_0.15_AsSb must be very different to those seen in more conventional avalanching materials like InP and InAlAs.

**Figure 4:**
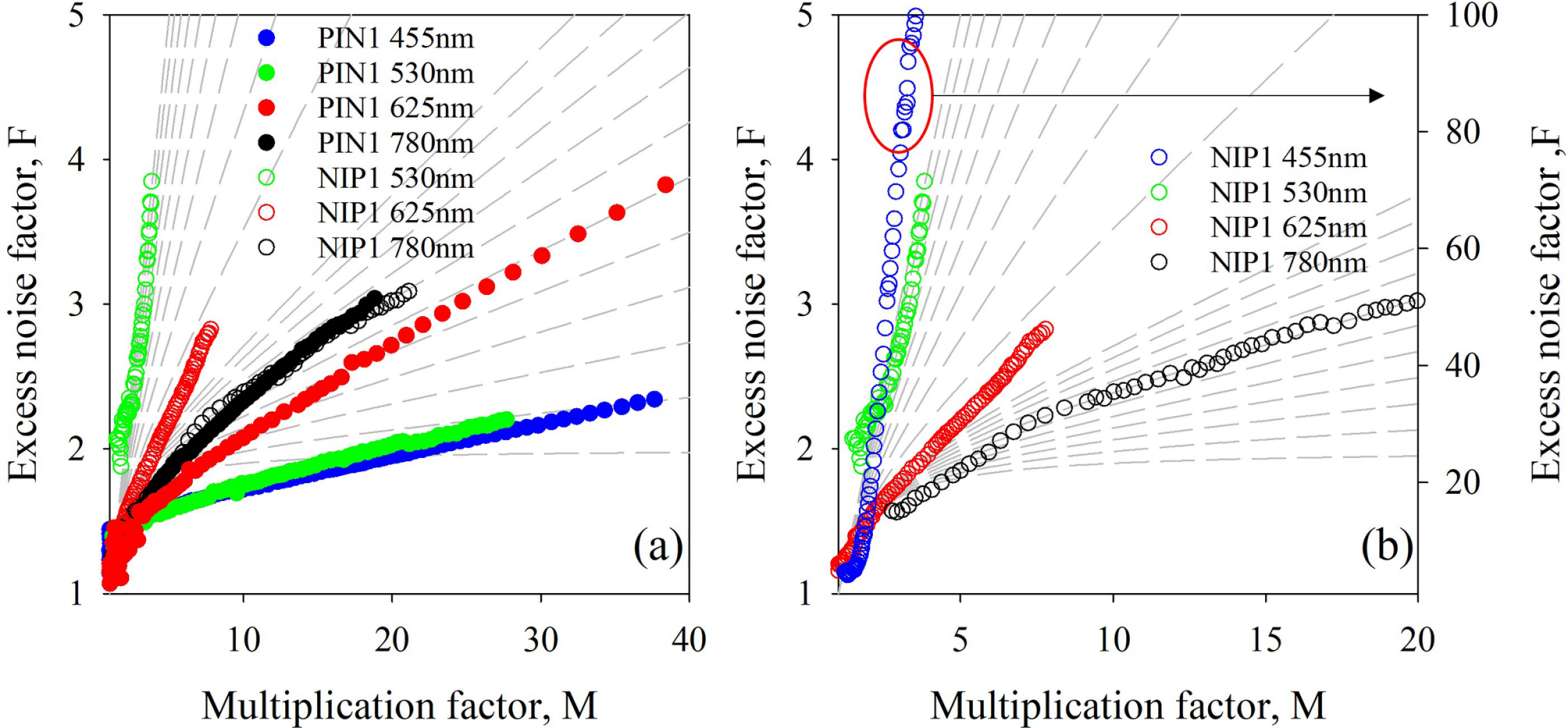
(**a**) Excess noise data for PIN1 under pure and mixed injection conditions and NIP1 under mixed injection conditions. Dashed lines indicate the noise predicted by McIntyre’s local model for an effective *k* of 0 to 0.1 in steps of 0.01 and 0.1 to 1 in steps of 0.1. (**b**) Excess noise data for NIP1, shown on two different y-axis scales to include results for NIP1 under pure hole injection conditions. Dashed lines indicate the noise predicted by McIntyre’s local model for an effective *k* of 0 to 0.1 in steps of 0.01 and 0.1 to 1 in steps of 0.1.

Figure 4 shows excess noise data for PIN1 and NIP1 under varying injection conditions. Data for pure electron injection and mixed injection for both samples is shown in Fig. 4a. Noise increases with increasingly mixed injection in the PIN structure and decreases with increasingly mixed injection in the NIP structure. The excess noise factor for uniformly mixed injection, under illumination at 780 nm, was similar in the PIN and NIP structures, and approximately equivalent to that predicted by McIntyre’s local model for an effective *k* of 0.06. The excess noise factor for pure hole injection conditions, produced using 455-nm light on the NIP structure, was extremely high—approximately equivalent to an effective *k* of 50. This data is shown in Fig. 4b with a different y-axis scale. This is equivalent to an excess noise factor of 100 at a multiplication factor of approximately 3.7. Having a slightly mixed injection condition using a wavelength of 530 nm reduces the noise significantly (Fig. 4b) to that equivalent to an effective *k* of 1. The change seen in the PIN structures between illumination at 455 and 530 nm is almost negligible by contrast since α >  > β in this alloy.

## Discussion

The large change in noise performance and multiplication between pure hole injection and slightly mixed injection in the NIP structure indicates the dominance of electron-initiated impact ionization events in this material system, as does the relatively small change with small amounts of mixed injection in the PIN structures. This suggests that accurate determination of the ionization behaviour in materials with a large difference in *α* and *β* require both PIN and NIP structures to be studied and indicates the importance of ensuring pure carrier injection measurements.

The excess noise data reported in Fig. 3 do not correspond to those predicted by the McIntyre equation using the ionization coefficients of Guo et al*.*^7^, shown by the solid coloured lines or with a hard threshold energy RPL model shown by dashed coloured lines in Fig. 3. Even in the thickest structure, PIN2, these models significantly overestimate the excess noise. This discrepancy between measured excess noise and that predicted by Eq. (1) has also been observed in AlAsSb^4,25^. The small *β*/*α* ratio in thick avalanching materials containing Sb has been attributed to a suppression of the hole impact ionization, caused by the increased spin–orbit splitting energy in the valence band due to the presence of a large group V atom^26^. It may also be related to its indirect band gap and large difference between the Γ and X energy gaps at this high aluminium alloy composition as observed in the Al_x_Ga_1−x_As and (Al_x_Ga_1−x_)_0.52_In_0.48_P^27^ material systems. However, the excess noise measured in this work is significantly lower (by about five times) than what would be expected due to the *β*/*α* ratio alone. To get good agreement with the experimental multiplication and excess noise data it was necessary to use a Weibull–Fréchet random path length (WF-RPL) model^25^. This model is similar to the commonly used hard-threshold RPL model^24^ but incorporates a Weibull–Fréchet (WF) distribution function for calculating the spatial probability distribution of carrier ionization. This allows the ionization threshold energy for each carrier type to be interpreted as ‘soft’, as opposed to a ‘hard’ threshold energy which approximates the ionization probability distribution as a displaced exponential decay function^12,28^. Both models differ from the conventional local model of impact ionization, which assumes that charge carriers have sufficient energy to impact ionize as soon as they are created. Ong et al.^25^ showed that the WF function can be used to replicate the high electric-field probability density functions (PDFs) of electron and hole ionization path lengths in GaAs obtained from full-band Monte Carlo simulation, and thereby predict the multiplication and excess noise behaviour in devices. They also showed that a WF-RPL model was needed to model the multiplication and excess noise in AlAs_0.56_Sb_0.44_ PIN diodes accurately.

**Figure 5:**
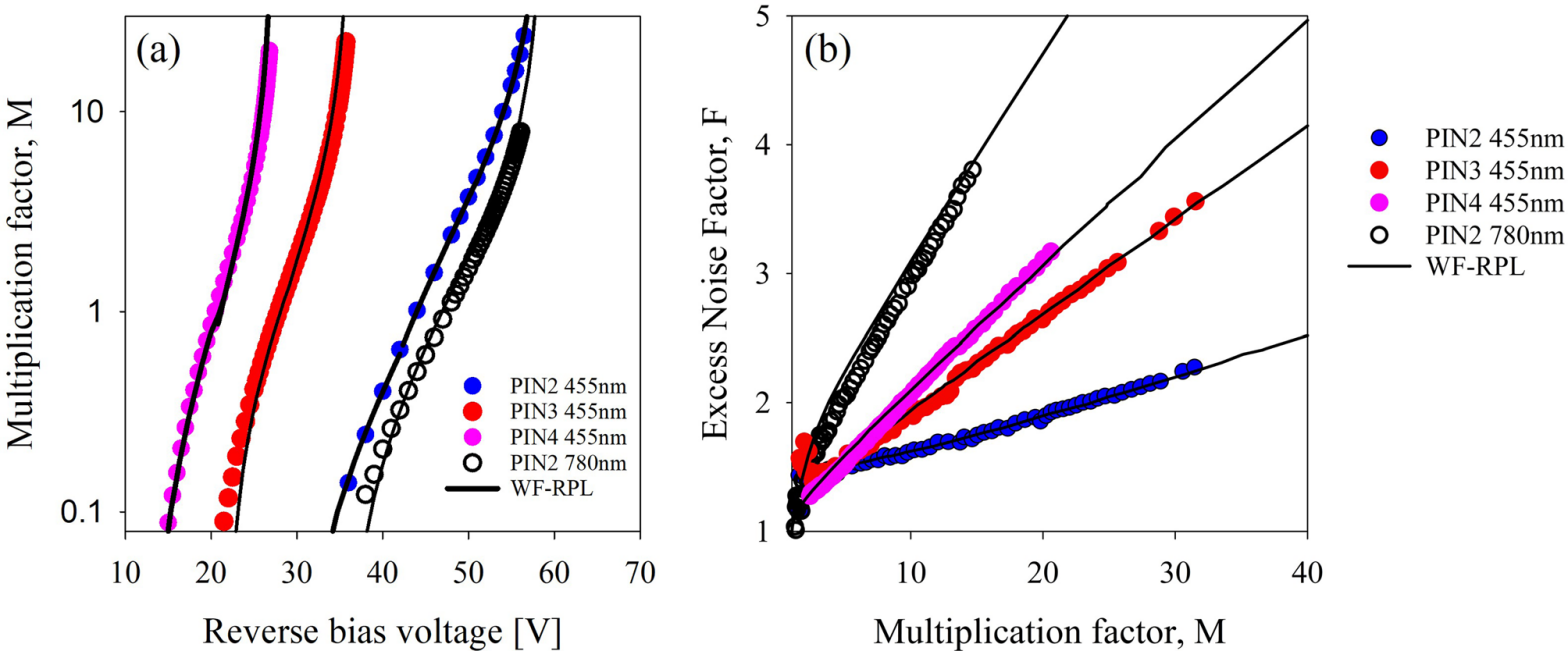
(**a**) Multiplication and (**b**) excess noise data for PIN2, PIN3, and PIN4 under pure electron injection conditions, shown with simulated data for these structures produced, using the ionization coefficients of Guo et al.^7^ and by a WF-RPL model using the parameters listed in Table 2. Also shown in (**a**,**b**) is the experimental and modelled multiplication and excess noise obtained under 780-nm illumination (open black circles).

**Figure 6:**
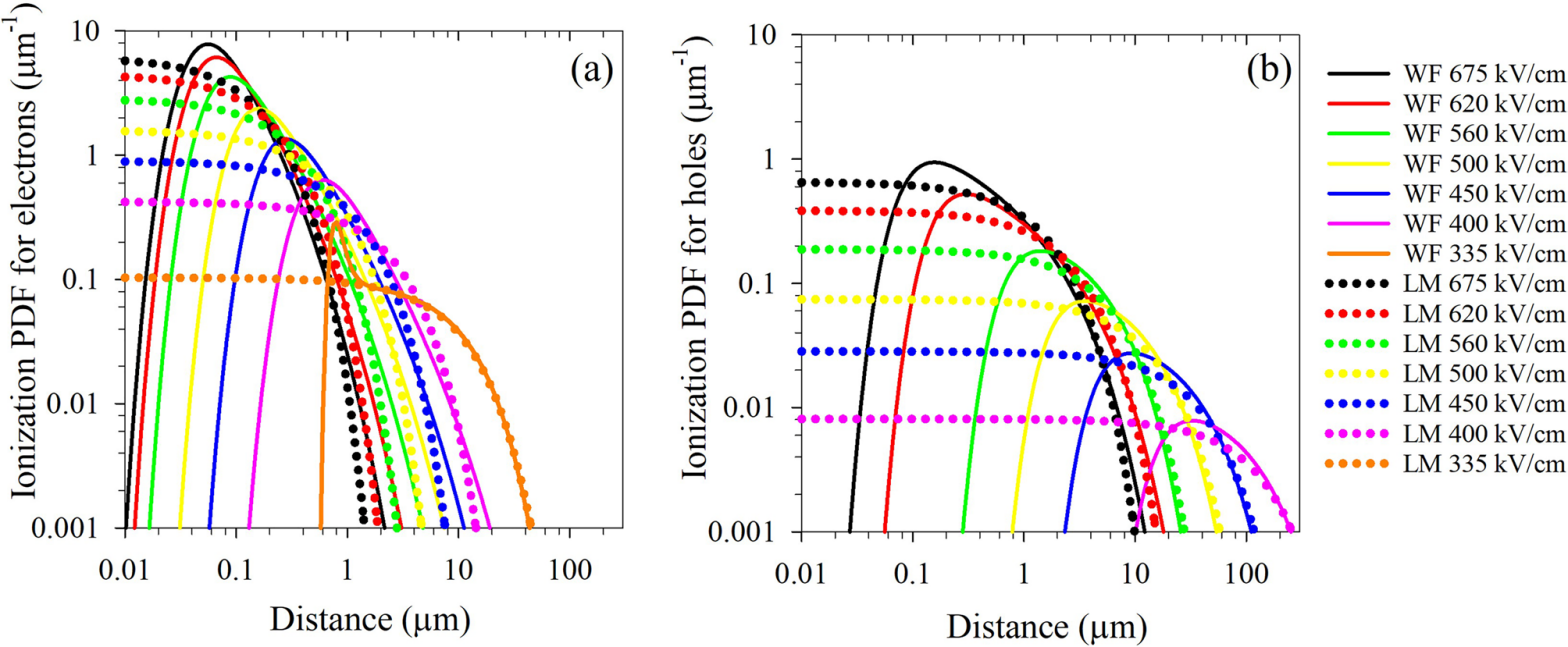
Weibull-Fréchet ionization probability density functions (PDFs) for (**a**) electrons and (**b**) holes at the breakdown fields of PIN2 and PIN4, as a function of distance travelled by a carrier through the high-field region. The PDFs used by a local model are shown for comparison.

Figure 5 shows fitted multiplication and excess noise data under pure electron injection for the RA PIN structures (PIN2, PIN3, and PIN4) from a WF-RPL model together with the measured data. The WF-RPL model assumes that the electric field strength is uniform throughout the high-field region of the device, and the low background doping in these structures means that this assumption is valid. The WF function used is a four-parameter model given by Eq. (2)^25^:

2 $$\begin{aligned} P\left( x \right) & = abcd^{c} x^{ - c - 1} {\text{exp}}\left[ { - b\left( \frac{d}{x} \right)^{c} } \right] \times \left\{ {1 - {\text{exp}}\left[ { - \left( \frac{d}{x} \right)^{c} } \right]} \right\}^{ - b - 1} \\ & \quad \times {\text{exp}}\left( { - a\left\{ {{\text{exp}}\left[ {\left( \frac{d}{x} \right)^{c} } \right] - 1} \right\}^{ - b} } \right) \\ \end{aligned}$$

where *d* is a scale parameter representing the mean ionization path length, < *l*_*e*_ >  = 1/*α*, or < *l*_*h*_ >  = 1/*β*, for electrons and holes respectively. *a*, *b*, and *c* are shape parameters representing the different features of the WF distribution. The values of these coefficients vary depending on electric field, and the values used are given in Table 2. These values have been determined empirically using the ionization coefficients of Guo et al*.*^7^ and by fitting to the measured multiplication and noise data reported here. The WF-RPLs could also replicate the multiplication and excess noise in PIN2 obtained with 780-nm illumination, when carriers are uniformly generated within the multiplication region. To get the best fit, as shown in Fig. 5, the contribution of electrons from the 300-nm *p*^+^ AlGaAsSb layer to the overall multiplication process had to be included. The WF-PDFs corresponding to a range of electric-fields for electrons (335–675 kV/cm) and holes (400–675 kV/cm) used in the modelling are shown in Fig. 6, in comparison with the exponential PDFs that would be used by a local model. The electron WF-PDF at 335 kV/cm shows a peaked distribution where, after an initial dead space, the ionization probability increases rapidly and then decreases rapidly before decreasing more gradually at a slower rate. This ‘peaked’ shape of this PDF results in a significant disparity in the shape parameters (*a*, *b* and *c*) compared to the values used for other electric field conditions for the same carrier, as presented in Table 2. A similarly peaked PDF was necessary to simulate the electron multiplication and excess noise in AlAsSb^25^. Both AlAsSb and AlGaAsSb material systems may be showing something akin to the behaviour predicted by Ridley’s ‘lucky-drift’ model^29^ of impact ionization at this relatively low electric-field and may explain the *F* < 2 experimental results. No experimental data could be obtained for *β* at such a low field in this material system.

**Table 2:** Parameters used for Weibull-Fréchet distribution functions.

Carrier type	Electric field (kV/cm)	*a*	*b*	*c*	*d*
Electron	335	0.1	0.16	6.5	1/*α*
	400	1.72	0.48	1.1	1/*α*
	450	1.72	0.48	1.1	1/*α*
	500	1.72	0.48	1.1	1/*α*
	560	1.72	0.48	1.1	1/*α*
	620	1.9	0.5	1.1	1/*α*
	675	2.1	0.52	1.1	1/*α*
Hole	400	0.5	0.8	1.22	1/*β*
	450	0.5	0.8	1.22	1/*β*
	500	0.5	0.8	1.22	1/*β*
	560	0.5	0.8	1.22	1/*β*
	620	0.38	0.68	1.17	1/*β*
	675	0.36	0.67	1.16	1/*β*

**Figure 7:**
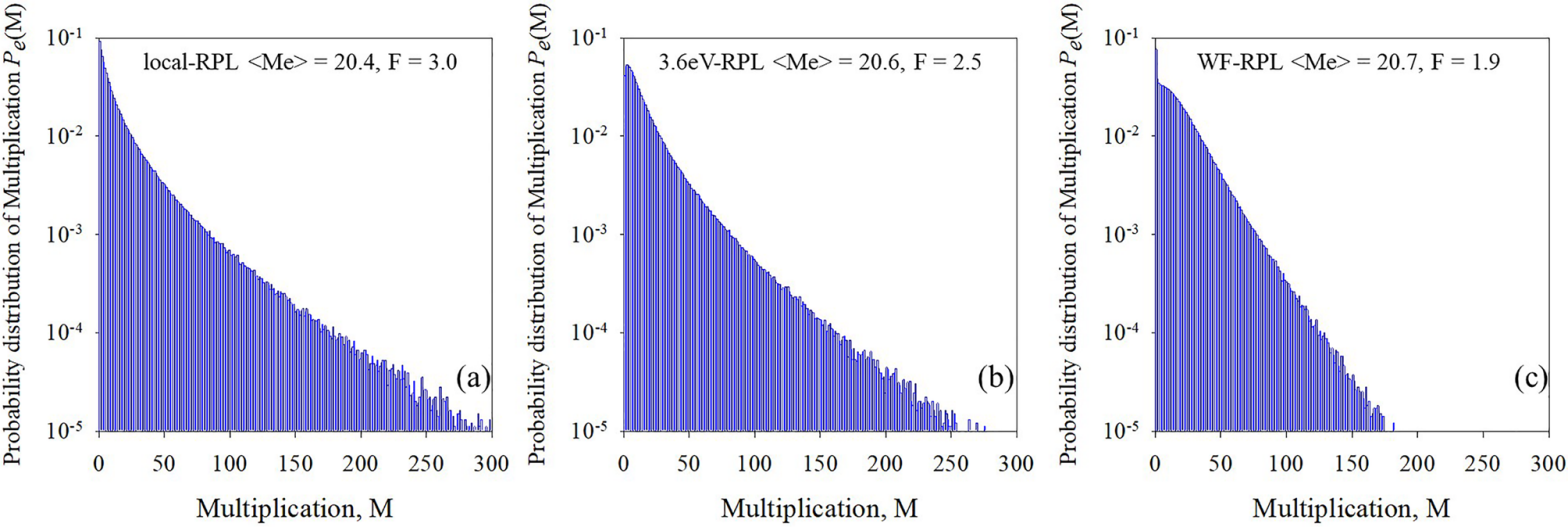
Probability distribution of multiplication Pe(M) of (**a**) local model, (**b**) RPL model with ‘hard’ dead space and (**c**) WF-RPL model. Despite the mean multiplication being fairly similar in all three cases, the shape of the WF-PDF results in a lower excess noise.

Since *F* is defined as:

3 $$F = \frac{{\left\langle {M^{2} } \right\rangle }}{{M^{2} }}$$

it is instructive to show the probability distribution of multiplication (*P*_e_(*M*)) in PIN2 for a mean electron-initiated multiplication of ~ 20 with a local model, an RPL model with a hard dead space and a WF-RPL model in Fig. 7. Figure 7a shows that *P*_e_(*M*) for a local model has a decaying probability that goes out to *M* values of ~ 300 while the presence of the hard dead space limits the range of *M* values to ~ 270 (Fig. 7b), thereby reducing the *F* from 3.02 to 2.62. The WF PDF however reduces the range of the maximum *M* to ~ 170 (Fig. 7c), thereby dramatically reducing the F further to 1.92.

The WF PDFs shown in Fig. 6 are only strictly valid for the case of a constant electric-field. Recent experimental work^20^ and modelling^21^ suggests that in the presence of a varying electric-field, the excess noise may well be reduced further and this may be due to variations in the shape of the WF PDF. More experimental work needs to be undertaken to explain these results. Other Sb containing alloys may also exhibit similar behaviour to that seen in Al_0.85_Ga_0.15_AsSb. Yuan et al.^30^ reported that thick InAlAsSb grown lattice matched on GaSb has a *β/α* ionization coefficient ratio of 0.14 while the* F* does not follow Eq. (1) and is significantly lower than would be expected by its *k* value. Other compositions of Al_x_Ga_1−x_As_0.56_Sb_0.44_ may also show similar a reduced *F* irrespective of their ionization coefficient ratio.

The relative reduction in *F* compared to that predicted by the ionization coefficients appears to be larger for thicker structures, contrary to the behaviour seen in materials like InP and InAlAs^22,23^. APDs with multiplication regions thicker than the maximum of 1020-nm reported here may well give even lower excess noise although possibly with a lower bandwidth and higher operating voltage.

## Data Availability

Data underlying the results presented in this paper are available from Prof. John David (j.p.david@sheffield.ac.uk) upon reasonable request.
